# Solid Phase Extraction and Determination of Tetracycline Using Gold Nanoparticles Stabilized in a Polymethacrylate Matrix

**DOI:** 10.3390/molecules30224458

**Published:** 2025-11-19

**Authors:** Nadezhda V. Saranchina, Daria E. Kuznetsova, Nataliya A. Gavrilenko, Mikhail A. Gavrilenko

**Affiliations:** 1 Department of Analytical Chemistry, Tomsk State University, 634050 Tomsk, Russia; 2 Department of Chemistry, Surgut State University, 628403 Surgut, Russia

**Keywords:** tetracycline, polymethacrylate matrix, solid phase extraction, solid phase spectrophotometry, solid phase fluorimetry, colorimetry

## Abstract

A polymethacrylate matrix (PMM) is proposed for the solid-phase extraction and determination of tetracycline (TC). The study of the influence of medium acidity, temperature, and contact time on the extraction of tetracycline by PMM showed that tetracycline is extracted by the matrix in the form of a singly charged anion H_2_TC^−^, within the pH range of 8.9–9.7, with distribution coefficients reaching (5–6) × 10^3^ mL/g. Following the extraction process using PMM and PMM-Au^0^, the direct determination of tetracycline in the solid phase is possible without an elution step. This is achieved by using as the analytical signal both the intrinsic absorption and the instrumentally measured peak area of the anionic form of tetracycline, H_2_TC^−^, in the matrix, with detection limits of 0.03 and 0.01 mg/L, respectively, and the fluorescence of tetracycline in PMM and PMM-Au^0^, with detection limits of 0.001 and 0.005 mg/L, respectively. The applicability of the digital colorimetry method for the quantitative determination of tetracycline based on its fluorescence in the solid phase is demonstrated. Methodologies for the determination of tetracycline using PMM and PMM-Au^0^ were developed and tested in the analysis of river and bottled water samples, biological fluid, as well as honey and milk samples.

## 1. Introduction

Tetracycline antibiotics are widely used today due to their low cost and broad spectrum of action. Tetracyclines are used for the treatment and prevention of infectious diseases in humans; they are actively used in veterinary medicine for the prevention and treatment of infectious diseases in animals, in feed production for rapid growth and weight gain of animals, and also in agriculture [1,2]. Furthermore, antibiotics are used in the food industry, including in the production of bottled water [3]. The widespread use of tetracyclines and non-compliance with the dosages of these drugs leads to their presence in food products of animal and plant origin, and in environmental objects: soils, surface and groundwater, and drinking water, where they can enter with wastewater from pharmaceutical and agricultural enterprises [4,5]. Accumulation in the body as a result of systematic intake of tetracycline can lead to a number of diseases: allergic reactions, metabolic disorders, dysbiosis, and can also cause bacterial resistance to the antibiotic. Consumption of drinking water containing even microquantities of antibiotics significantly accelerates the development of resistance [6]. To protect human health and ensure food safety, regulations controlling the quality of food products have been introduced [7]. Therefore, it is important to ensure proper quality control of food products and environmental objects containing residual amounts of antibiotics. Analysis of human urine allows for the assessment of the level of exposure to antibiotics ingested through food or water, and also plays an important role in clinical practice. In this regard, the development of highly sensitive, selective, and rapid methods for the determination of tetracycline in complex matrices is an urgent task in analytical chemistry.

Various methods are used for the determination of tetracycline, each having its own advantages and disadvantages. One of the most accurate and selective methods is chromatographic–mass spectrometric methods (HPLC-MS, LC-MS/MS); however, these methods require expensive equipment, highly qualified operators, and complex sample preparation, which limits their use in routine analysis, especially in small laboratories [8,9,10,11,12,13,14]. Immunochemical methods, despite their high sensitivity and rapidity, depend on the quality of the reagents used and the analysis conditions [15]. Some of the most accessible and easy-to-use are optical methods [16], such as spectrophotometry [1,17,18] and fluorimetry [19,20,21,22,23,24]. However, their main limitation in determining trace amounts of analytes in complex matrices (biological fluids, food products, environmental objects) is their low sensitivity and strong influence of matrix effects, which necessitates preliminary concentration and isolation of the analyte from complex media.

Among the many concentration methods, solid-phase extraction is one of the most effective, reproducible, and convenient for automation approaches [25,26], as well as for creating hybrid methods for substance determination combining concentration stages in the form of solid-phase extraction and detection of the analyte directly in the solid phase using optical methods. This approach allows for the elimination of the laborious elution step, minimization of analyte losses, reduction in analysis time, and an increase in its sensitivity due to the combination of concentration and measurement operations. A key element of such hybrid systems is the solid-phase extractant material, which must not only efficiently extract the target analyte from the complex matrix but also possess optimal optical characteristics for direct spectrophotometric and fluorimetric measurements. In this context, polymethacrylate polymers are of exceptional interest due to their high optical transparency and low intrinsic background fluorescence. We propose using a polymethacrylate matrix (PMM) as a solid-phase extractant, obtained by radical block polymerization of methacrylate monomers in the presence of polyethylene glycol 400 (PEG 400), thus realizing a hydrophobic framework in the form of a polymethacrylate base, providing structural rigidity and analyte accumulation, and a hydrophilic base in the form of PEG400, ensuring diffusion of the analyte into the matrix volume [27,28]. One of the key advantages of tetracycline as an analysis object is its intrinsic absorption in the visible region of the spectrum and its ability to exhibit intrinsic fluorescence under certain conditions [29]. This opens up the possibility for creating direct, label-free spectrophotometric and fluorimetric methods, the sensitivity of which can be enhanced by using PMM for solid-phase extraction of tetracycline (TC) and its subsequent determination in the solid phase by spectrophotometry and fluorimetry. For the targeted enhancement of the analytical signal of TC in PMM, gold nanoparticles (PMM-Au^0^) possessing surface plasmon resonance properties [5,30,31,32] were immobilized, and their introduction into PMM is intended to enhance interaction with TC during its extraction into PMM-Au^0^, which is promising for solving the problems of monitoring trace amounts of TC in biological fluids, food products, and environmental objects.

It can be stated that the determination using solid-phase extraction is more sensitive than that for aqueous tetracycline solutions. Aqueous tetracycline solutions possess low fluorescence quantum yields of ~10^−3^, which makes direct fluorimetry ineffective for the determination of trace quantities. This is due to rapid non-radiative processes in a protic medium, and enhancing fluorescence requires special conditions, such as organic solvents [29]. In the PMM polymer matrix, fluorescence enhancement is observed, which is due to both the concentration of the analyte and the influence of a less polar and protonated environment. This reduces the probability of quenching and promotes an increase in the efficiency of fluorescent emission.

Thus, the aim of the present work was to develop and study a hybrid method for the determination of TC, based on its solid-phase extraction into PMM and PMM-Au^0^ with subsequent direct quantitative measurement in the solid phase using spectrophotometry and fluorimetry.

## 2. Results and Discussion

### 2.1. Spectrophotometric TC Determination Using PMM

**Figure 1:**
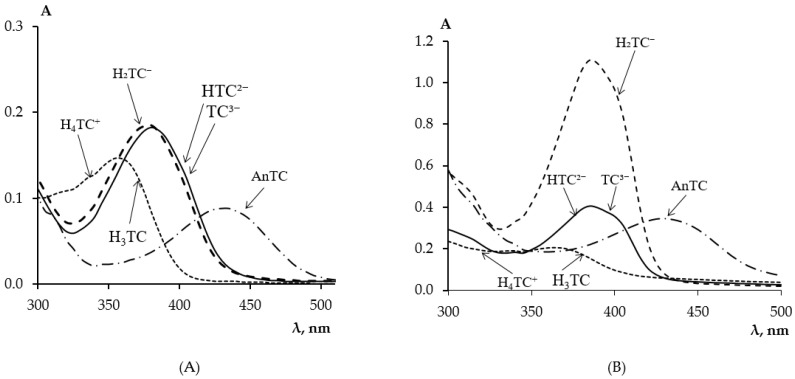
Absorption spectra of TC in aqueous solutions (**A**) and in PMM (**B**) after its contact with a TC solution at different pH values: 1–0.5; 2–3.2; 3–9.3; 4–12.0; (*C*_TC_ = 5 mg/L, *t*_contact_ = 60 min).

Tetracycline is an amphoteric molecule with multiple functional groups and, depending on pH, can exist in aqueous solutions in five ionic forms [33,34]. Figure 1A,B show the absorption spectra of TC in aqueous solutions with different pH values and in PMM after its extraction by the matrix from these solutions, respectively.

In a strong acidic medium at pH ≈ 0.5, tetracyclines are converted to anhydrotetracyclines, AnTC, according to the scheme presented in work [35], and exhibit a dark yellow color with an absorption maximum at 440 nm [36]. In an acidic medium at pH < 3.2, TC is in the cationic form H_4_TC^+^ with an absorption maximum at a wavelength of 430 nm. When the medium acidity changes from acidic to neutral 1.8 < pH < 7.6, TC transitions from H_4_TC^+^ to the zwitterionic form H_3_TC with an absorption maximum at a wavelength of 355 nm. In a weakly alkaline medium, deprotonation of the molecule continues, leading to the formation of the singly charged anionic form H_2_TC^−^ 7.6 < pH < 9.6 with an absorption maximum at 380 nm. A further increase in pH promotes the deprotonation of the second phenolic hydroxyl of the phenolic diketone part, leading to the formation of the doubly charged anion HTC^2−^ 9.6 < pH < 12 and at pH > 12, the triply charged anion TC^3−^ is formed without changing the absorption spectrum characteristic of the anionic form H_2_TC^−^.

From the absorption spectra of TC in PMM (Figure 1B) after its solid-phase extraction from solutions of different acidity, it is evident that the pH of the solution affects the extraction of TC by the matrix from the solution. Table 1 presents the absorption maxima of the ionic forms of TC in aqueous solutions and in PMM depending on the medium acidity.

For the quantitative characterization of the extraction process of TC from solutions with different pH values, its distribution coefficients were determined, presented in Table 2. As can be seen, the best extraction of TC occurs from solutions with pH from 8.9 to 9.7 in the form H_2_TC^−^, with a small shoulder appearing on the right side of the TC absorption spectrum in PMM compared to its solution. This may be due to the coordination of TC by the calcium ion present in PMM as a cross-linking agent—calcium methacrylate. According to [37], TC forms a complex with the calcium ion in an aqueous solution in a 1:1 ratio at pH 7.5–11.7, and the absorption spectrum of this complex is identical to the absorption spectrum of TC in PMM. However, it should be noted that increasing the pH of the TC solution above 9.7 led to a decrease in TC extraction by PMM. Therefore, all further studies on the extraction of TC by the matrix and its determination by solid-phase spectrophotometry were carried out at pH 9.18 in a borate buffer medium.

The optical density at the maximum absorption of TC in PMM at a wavelength of 385 nm (*A*_385_) or the absolute change in optical density, Δ*A*_385_ = *A* − *A*_0_, where *A* and *A*_0_ are the absorbances of the PMM at 385 nm after contact with the solution in the presence and absence of the TC, respectively, was used as the analytical signal.

**Figure 2:**
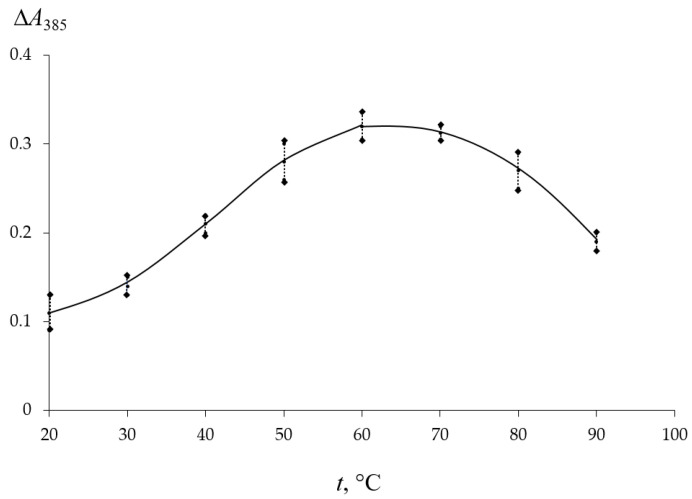
Influence of temperature on the analytical signal ∆*A*_385_ after contact of PMM with TC solution *C*_TC_ = 2 mg/L for 60 min.

The influence of the temperature of the TC solution on its solid phase extraction was assessed by the magnitude of the analytical signal Δ*A*_385_ after contact of the matrix with the TC solution upon heating in the range of 20–90 °C. From the dependence presented in Figure 2, it can be seen that the analytical signal is maximal after contact of PMM with antibiotic solutions at a temperature of 60–70 °C. With a further increase in temperature, a decrease in Δ*A*_385_ is observed. This is likely due to the degradation of the analyte at high temperatures. Based on the presented results, the extraction of TC by the matrix was carried out by heating the solutions to 60 °C.

The influence of the contact time of PMM with the TC solution on its extraction under optimal conditions was investigated by constructing dependencies of the analytical signal ∆*A*_385_ on the antibiotic concentration in the solution at different contact times. To increase the sensitivity of TC determination using PMM under acceptable conditions, the possibility of using the instrumentally measured peak area, bounded by the peak contour and its base—the segment connecting the start and end of the absorption maximum of the anionic form of TC H_2_TC^−^ in the matrix at 385 nm, as the analytical signal was considered. This approach is associated with minimizing the influence of the intrinsic background absorption of PMM on the magnitude of the analytical signal. Table 3 presents the parameters of the calibration dependencies and the analytical characteristics of TC determination. From the given data, it can be seen that with an increase in the contact time of PMM with the TC solution, the sensitivity of the analyte determination increases, and its detection limit, calculated by the 3*s*-criterion, decreases. The use of the instrumentally measured peak area as the analytical signal allows for an increase in the sensitivity of TC determination. Figure S1 shows the absorption spectra of TC in the matrix after contact of the matrices with TC solutions of different concentrations at pH 9.18 and a contact time of 90 min.

### 2.2. TC Determination by Its Intrinsic Fluorescence Using PMM and PMM-Au^0^

A promising alternative to the spectrophotometric determination of tetracyclines using PMM is the measurement of the direct fluorescence of the antibiotic in the solid phase. This approach is based on the ability of tetracyclines to fluoresce under UV irradiation without the introduction of additional reagents, as well as on the fact that the luminescence method generally surpasses spectrophotometry in sensitivity. Although aqueous solutions of TC are characterized by weak fluorescence, its extraction by PMM allows for the concentration of the antibiotic in the solid phase, which significantly increases the sensitivity of determination. It is important to note that after the extraction of TC, the PMM plate remains transparent, ensuring high accuracy of analytical signal measurements.

**Figure 3:**
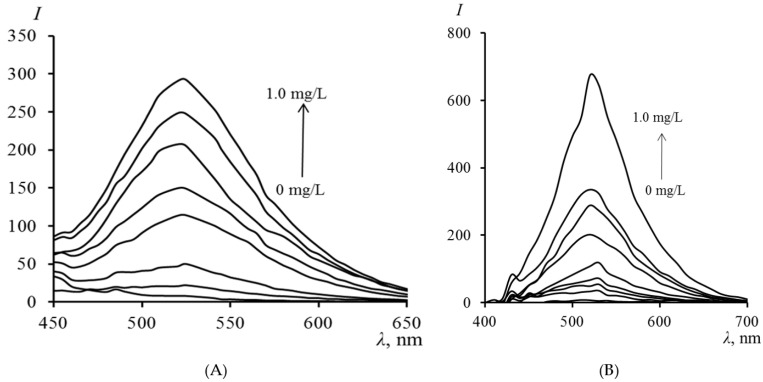
Fluorescence spectra of TC in PMM (**A**) and PMM-Au^0^ (**B**) after contact with solutions of different concentrations (excitation at 390 nm).

Figure 3 presents the fluorescence spectra of TC in PMM after contact of the matrix with a TC solution. It can be seen from the figure that the fluorescence intensity in the matrix regularly increases with an increase in the concentration of TC in the analyzed solution. The fluorescence maximum of TC in the PMM (520 nm) coincides with its fluorescence in organic solvents such as acetonitrile and dimethyl sulfoxide [29]. The coincidence of the fluorescence maximum of TC in PMM with the data for aprotic organic solvents indicates that the matrix provides TC with a similar environment that suppresses intermolecular proton transfer, characteristic of water. Additionally, the “rigidity” of the polymer environment restricts the mobility of the TC molecule, which may suppress non-radiative processes and likely leads to a higher fluorescence quantum yield compared to an aqueous solution.

The promise of using the direct fluorescence of TC in PMM lies not only in the high sensitivity of the method but also in the variability of instrumental detection. In addition to recording the signal on a standard fluorimeter, its fixation by digital colorimetry is possible, which corresponds to modern trends in analytical chemistry. Currently, there is a steady trend towards the development of portable control tools that allow for rapid, inexpensive, and express analysis directly at the sampling site. One of the most promising directions for this is digital colorimetry, the key advantages of which are the simplicity of hardware design and the possibility of using widely available digital photo equipment.

The ubiquitous distribution of smartphones equipped with high-quality cameras and software for image processing plays a special role in the miniaturization of such methods. This opens up opportunities for creating mobile methods that are not inferior in accuracy to procedures using stationary equipment [32].

**Figure 4:**
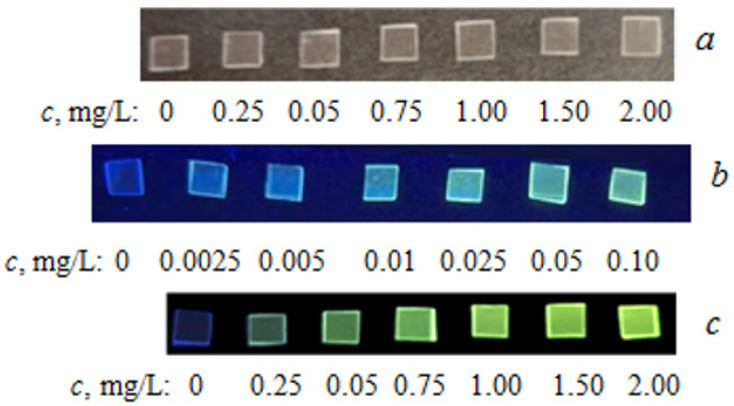
Photographs of PMM samples after contact with TC solutions of different concentrations: (**a**)—without irradiation; (**b**,**c**)—upon irradiation with UV light 365 nm.

The extraction of TC from the analyzed solutions by PMM was carried out under optimal conditions previously selected for spectrophotometric determination. After completion of the extraction, the plates were removed from the solution and irradiated with light at a wavelength of 365 nm to excite fluorescence. The TC content was found from the calibration dependence constructed under similar conditions. Table 4 presents the parameters of the calibration dependencies and the analytical characteristics of TC determination by its intrinsic fluorescence using PMM (Figure 4).

**Table 4:** Analytical characteristics of TC determination by its intrinsic fluorescence using PMM and PMM-Au^0^ (*t* = 60 min).

Extractant	Equation	Color	*R*	AR, mg/L	LOD, mg/L
PMM	∆*E* = 1150*c*	Blue (Figure 4b)	0.995	0.0025–0.1000	0.001
	∆*E* = 74*c* + 158	Yellow-Green (Figure 4c)	0.994	0.25–2.00	0.21
PMM-Au^0^	Δ*E* = 10,571*c*	Blue (Figure 5)	0.992	0.001–0.010	0.0005
	∆*E* = 469*c* + 142	Yellow-Green (Figure 5)	0.994	0.025–0.100	0.012

**Figure 5:**
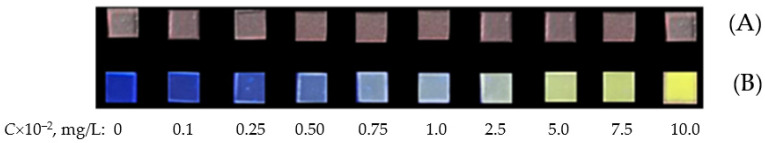
Photographs of PMM-Au^0^ samples after contact with TC solutions of different concentrations: (**A**)—without irradiation; (**B**)—upon irradiation with UV light (365 nm).

Also, the interaction of TC with a composite material based on polymethylmethacrylate containing gold nanoparticles (PMM-Au^0^) was investigated in the work, since it is known that for fluorophores located near the surface of metallic nanostructures, an increase in quantum yield is possible [38]. Depending on the nature of the reducing agent used in the synthesis, samples with different optical properties were obtained: upon reduction with sodium borohydride, the plates had a red color with a plasmon absorption maximum at 530 nm, and when using ascorbic acid, they had a gray-violet color with a maximum at 575 nm (Figure S2).

After the extraction of TC by PMM-Au^0^ and subsequent irradiation with UV light, intense yellow fluorescence of the antibiotic was observed. A key result is the significant enhancement of the fluorescent signal in the presence of gold nanoparticles compared to pure PMM, which is explained by the metal-enhanced fluorescence effect [39,40,41,42,43].

During preliminary experiments, it was found that PMM-Au^0^ samples synthesized using ascorbic acid exhibit fluorescence in the blue region of the visible spectrum under UV irradiation, and the signal intensity increases during sample storage [44]. This background fluorescence significantly hinders the registration of the TC signal after its extraction. In this regard, for all subsequent studies, PMM-Au^0^ samples (Figure S3) obtained using sodium borohydride were used, which did not possess interfering intrinsic fluorescence.

**Figure 6:**
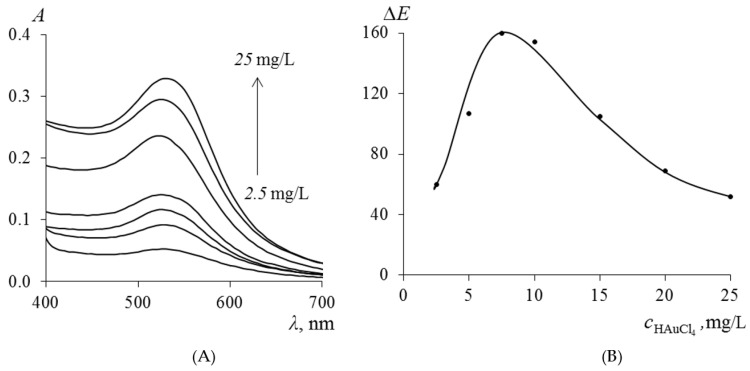
Absorption spectra of Au NPs synthesized in PMM (**A**) and dependence of the analytical signal ∆*E* on the concentration of Au NPs in PMM after contact of PMM-Au^0^ with a 0.1 mg/L TC solution (**B**).

The influence of the concentration of gold nanoparticles (Au NPs) in PMM after the extraction of TC on the magnitude of the ∆*E* was varied by changing the concentration of the initial HAuCl_4_ solution in the range from 2.5 to 25.0 mg/L at the stage of immobilization of Au(III) ions. The absorption spectra of the obtained samples, presented in Figure 6A, demonstrate that with an increase in the concentration of HAuCl_4_, the intensity of the plasmon resonance maximum at 530 nm increases, which directly indicates an increase in the concentration of synthesized Au NPs in the polymer matrix. According to the data in Figure 6B, the ∆E reaches a maximum when using HAuCl_4_ solutions with a concentration of 5.0–7.5 mg/L for the synthesis of PMM-Au^0^.

The extraction of TC from the analyzed solutions by PMM-Au^0^ was carried out under optimal conditions previously selected for spectrophotometric determination. After completion of the extraction, the plates were removed from the solution and irradiated with light at a wavelength of 365 nm to excite fluorescence. Table 4 presents the parameters of the calibration dependencies and the analytical characteristics of TC determination by its intrinsic fluorescence using PMM-Au^0^. Figure 6 shows photographs of PMM-Au^0^ after contact with TC solutions of different concentrations without and upon irradiation with UV light. It can be seen from the figure that the intensity of yellow fluorescence in the matrix regularly increases with an increase in the concentration of TC in the analyzed solution. Based on the conducted research, methodologies for the determination of TC using PMM and PMM-Au^0^ by solid-phase spectrophotometry and solid-phase colorimetry were developed and tested on real samples.

### 2.3. TC Determination Procedure

The developed methodology was tested on real samples of human urine, river and bottled drinking water samples, as well as on milk and honey. When determining TC in bottled drinking and river waters, preliminary sample preparation of the analyzed samples was not required. In the case of biological and food analysis objects, preliminary sample preparation was carried out to remove proteins. When determining TC in urine, 10 mL of the analyzed sample was placed in a 15 mL plastic tube, 0.1 mL of a 20% trichloroacetic acid solution was added, mixed on a rotary shaker for 5 min, and then centrifuged for 5 min at 3000 rpm. The supernatant was transferred to another tube. For milk sample preparation, 10 g of the sample was placed in a 50 mL centrifuge tube, 0.4 g of ethylenediaminetetraacetic acid (EDTA), and 0.3 mL of concentrated glacial acetic acid were added, thermostated in a water bath for 10 min at 50 °C, and then centrifuged for 5 min at 4000 rpm. The supernatant was filtered through a paper filter. When analyzing honey, 20 g of the sample was placed in a 50 mL centrifuge tube, brought to a volume of 45 cm^3^ with distilled water, and stirred until complete homogenization. The sample was thermostated in a water bath for 10 min at a temperature of 50 °C, then centrifuged for 10 min at g-force 1431 g. The resulting supernatant was filtered through a paper filter into a conical flask.

When analyzing samples for TC content by the standard solutions method, a 0.5–3.6 mL aliquot of the analyzed samples was placed in a 5 mL tube, 0.4 mL of borate buffer was added, diluted to a volume of 4 cm^3^ with distilled water, and a PMM or PMM-Au^0^ plate was added. The contents of the tube were stirred for 60–90 min at a temperature of 60 °C, the samples were removed from the tube, and the analytical signal was measured. The TC content in the investigated samples was found from the calibration dependencies constructed under similar conditions (Table 3 and Table 4).

When determining TC in analysis objects using calibration dependencies constructed by the standard addition method, the procedure described above was performed. The difference was that additionally, solutions of reference samples with the content of 0.02–0.30 mL of a working TC solution with a concentration of 10 mg/L and 0.01–0.05 mL with a concentration of 1 mg/L were prepared. Then, a graph of the dependence of the analytical signal on the concentration of the additive was plotted, and the obtained straight line was extrapolated to the intersection with the concentration axis.

The accuracy of the analysis was checked by the standard addition method. The TC additive was introduced into the samples of human urine, milk, and honey at the first stage of sample preparation. The results of TC determination in the analyzed objects are given in Table 5. The relative standard deviation of the analysis results does not exceed 19%. The quantitative determination of additions of the standard TC solution in the analysis objects is determined with sufficient accuracy (Table S2).

As a result, it is shown that PMM and PMM-Au^0^ can be used for direct extraction of TC, followed by solid-phase spectrophotometric and solid-phase colorimetric determination in the range of TC concentrations 0.001–1.50 mg/L with a detection limit of 0.0005 mg/L with a sample volume of 4 mL. The proposed methods are simple in execution and comply with the principles of “green” chemistry, since they exclude the stage of elution and the use of toxic organic solvents, due to the unique properties of PMM, which allow direct measurement of the analyte in the solid phase.

## 3. Materials and Methods

### 3.1. Preparation of the PMM

The description is given in Section S1.

### 3.2. Preparation of Solutions

The description is given in Section S2.

### 3.3. Preparation of Gold Nanoparticles in PMM

Stable gold nanoparticles Au^0^ were formed directly in the transparent PMM by in situ synthesis through the reduction of Au(III) ions. At the first stage, immobilization of Au(III) into the matrix (PMM-Au^3+^) from a HAuCl_4_ solution with a concentration of 2.5–25.0 mg/L was carried out. For this, 20–60 PMM plates were stirred on a rotator with 5–15 mL of the solution for 5 min. At the second stage, the synthesis of nanoparticles in situ was initiated by immersing the PMM-Au^3+^ matrix into a reducing agent solution. Reductants of varying strength were used: a 1% NaBH_4_ solution (reduction time 1 min) and a 5% ascorbic acid solution (reduction time 5 min).

### 3.4. Experimental Methodology

The extraction of TC by PMM and PMM-Au^0^ was studied under various conditions. For this, a TC solution, a solution for creating the necessary pH value, with different concentrations of reacting substances, were placed into a 5 mL graduated tube, diluted to a volume of 4 cm^3^ with distilled water, and PMM was added. The contents of the tube were stirred for 15–120 min at a temperature of 20–90 °C, and the absorption spectra were recorded or the optical density at the maximum of the TC absorption band in the matrix was measured.

The efficiency of extraction recovery was assessed by the distribution coefficients (*D*), which were calculated by the formula:

$$D=\left[\frac{\left(A_{0}-A\right)}{A}\right]\times \left(\frac{V}{m}\right),$$

where *A*_0_ and *A* are the optical densities of the TC solution before and after extraction;

*V* is the total volume of the solution, mL;

*m* is the mass of the PMM plate, g.

### 3.5. Instrumentation and Operating Parameters

A scanning spectrophotometer UV-1800 (Shimadzu, Nakagyo-ku, Japan) was used for the determination of absorption spectra and absorbance of the PMM and the solutions in the visible region. The optical characteristics of the matrices after the TC extraction process were measured relative to the initial PMM. Fluorescence spectra of the matrices were recorded using an Agilent Cary Eclipse spectrofluorimeter (Agilent, Waldbronn, Germany). Fluorescence excitation at 395 nm was carried out using a Wood’s lamp (Izmeritelnaya Tekhnika, Moscow, Russia). Fluorescent emission in the yellow-green region, 520 ± 5 nm, was recorded using a smartphone or an ultraviolet analytical recorder UVC-HD (PetroChem, Sankt-Peterburg, Russia). Color difference Δ*E* in the *RGB* system was used as the analytical signal, calculated by the formula: ∆*E* = ((*R* − *R*_0_)^2^ + (*G − G*_0_)^2^ + (*B − B*_0_)^2^)^1/2^, where *R*, *G*, *B*, *R*_0_, *G*_0_, and *B*_0_ are the intensity values of red, green, and blue colors of the analyzed and blank samples, respectively.

The electron microscopy (SEM) images were registered using a JEM-2100 (JEOL, Akishima-shi, Japan) microscope with a thermal field-emission cathode at an acceleration voltage of 200 kV. BioSan Multi Bio RS-24 (BioSan, Riga, Latvia) multi-rotator was used for solution mixing. The pH values of the solutions were measured using an I-160 laboratory-grade ion meter (Izmeritelnaya Tekhnika, Moscow, Russia) with silver chloride reference electrodes. For centrifuging real samples at the sample preparation stage, a Stegler CM-600S (NV-Lab, Moscow, Russia) laboratory centrifuge was used. ColourGrab V. 3.9.2. software was used to process the obtained images.

## 4. Conclusions

A new hybrid method for the determination of TC has been developed, based on its solid-phase extraction into a PMM and a composite based on it containing gold nanoparticles PMM-Au^0^, with subsequent direct quantitative measurement in the solid phase using spectrophotometry and fluorimetry. The method is characterized by high sensitivity, selectivity, and rapidity. The developed methodology was tested on real samples of human urine, river and bottled drinking water, as well as on milk and honey. The relative standard deviation of the analysis results does not exceed 19%. The developed method can be used for the determination of TC in complex matrices, including biological fluids, food products, and environmental objects.

## Figures and Tables

**Table 1:** Absorption maxima of ionic forms of TC in aqueous solution and in PMM after its contact with the TC solution.

Ionic Form of TC	pH of TC Solution	Absorption Maximum λ, nm	
		Water Solution	PMM
AnTC (1)	0.5	430	
H_4_TC^+^ (2)	1.8–7.0	355	365
H_3_TC (2)			
H_2_TC^−^ (3)	7.0–9.6	380	385
HTC^2−^ (4)	9.6–12.9		390
TC^3−^ (4)			

**Table 2:** Distribution coefficients of TC during its extraction by the matrix from solutions with different pH values (*n* = 3).

pH	0.5	1.8	2.2	3.2	4.2	5.9	6.4	7.0	8.2	8.6	8.9	9.1	9.3	9.7	11.0	12.9
D, mL/g	102	129	45	25	5	116	118	143	183	194	511	588	578	554	159	81

**Table 3:** Analytical characteristics of TC determination using PMM at different extraction times.

Extraction Time, min	Equation	AR, mg/L	LOD, mg/L	*r*
15	Δ*A* = 0.17*c*	0.5–5.0	0.30	0.9917
	*S* = 7.13*c*	0.2–5.0	0.10	0.9925
30	Δ*A* = 0.28*c*	0.3–3.0	0.10	0.9970
	*S* = 11.31*c*	0.1–3.0	0.05	0.9838
60	Δ*A* = 0.33*c*	0.1–2.0	0.06	0.9970
	*S* = 13.42*c*	0.05–2.0	0.03	0.9935
90	Δ*A* = 0.38*c*	0.05–1.5	0.03	0.9927
	*S* = 17.21*c*	0.03–1.5	0.01	0.9965
120	Δ*A* = 0.47*c*	0.04–1.5	0.02	0.9938
	*S* = 20.29*c*	0.02–1.5	0.01	0.9938

**Table 5:** Results of TC determination in the analyzed objects (*n* =3–5; *p* = 0.95).

Object	Extractant	Added, mg/L	Calibration Method	Analytical Signal	Found, mg/L	*s*_r_, %	Recovery, %
Wildflower Honey	PMM	0	Standard Solutions	∆*E*	<LOD	-	-
		0.1			(0.11 ± 0.03)	10	106
Drinking Water	PMM -Au^0^	0	Standard Solutions		<LOD	-	-
		0.010			0.011 ± 0.002	17	112
		0.030			0.029 ± 0.003	7	98
Drinking Milk		0	Standard Additions		<LOD	-	−
		0.1			(0.09 ± 0.03)	14	92
Natural Drinking Water	PMM	0	Standard Additions		<LOD	-	-
		0.050		Δ*A*	0.053 ± 0.007	6	106
				*S*	0.053 ± 0.006	8	106
		0.100		Δ*A*	0.09 ± 0.04	19	91
				*S*	0.095 ± 0.012	8	95
River Water		0			-	-	-
		0.050		Δ*A*	0.055 ± 0.017	13	110
				*S*	0.046 ± 0.006	5	92
		0.100		Δ*A*	0.12 ± 0.04	16	116
				*S*	0.095 ± 0.017	7	95
Human Urine		0	Standard Solutions		-	-	-
		15		Δ*A*	16 ± 6	15	109
				*S*	15 ±4	10	99
				∆*E*	16 ± 6	15	109
			Standard Additions	Δ*A*	17 ± 3	7	116
				*S*	16.2 ± 2.2	5	108
		35	Standard Solutions	Δ*A*	37 ± 11	12	105
				*S*	34 ±6	7	96
				∆*E*	34 ± 9	10	97
			Standard Additions	Δ*A*	34 ± 9	10	97
				*S*	37 ± 5	6	105

Note: ∆*E*—Color difference in the RGB system; Δ*A*—Absorption at 385 nm; *S*—Fluorescent maximum at 520 nm.

## Data Availability

Data are contained within the article.

## Supplementary Materials

The following supporting information can be downloaded at: https://www.mdpi.com/article/10.3390/molecules30224458/s1, Section S1. Preparation of the PMM. Section S2. Preparation of Solutions. Figure S1. Absorption spectra of TC in PMM after contact with solutions of different concentrations. Figure S2. Scheme for obtaining gold nanoparticles in PMM. Figure S3. SEM images of nanoparticle associates in PMM-Au^0^ (1,2) and their size distribution (3). Table S1. Analytical characteristics of PMM-Au^0^ sensors with different storage periods. Table S2. Comparison between our sensor and that of other jobs.
