# Supplementary material for: Solid Phase Extraction and Determination of Tetracycline Using Gold Nanoparticles Stabilized in a Polymethacrylate Matrix
Source: Molecules. 2025 Nov 19;30(22):4458. doi: 10.3390/molecules30224458 (PMC12655131; doi:10.3390/molecules30224458)
Supplement: Supplementary file 1 [file molecules-30-04458-s001.zip › molecules-3970252-supplementary.pdf]

Solid Phase Extraction and Determination of Tetracycline Using Gold Nanoparticles Stabilized in a Polymethacrylate Matrix

N. V. Saranchina, D. E. Kuznetsova, N. A. Gavrilenko and M. A. Gavrilenko *

S1. Preparation of the PMM

Polymethacrylate matrix is a transparent colorlessness polymer material containing carbonyl and carboxyl groups providing for solid-phase extraction both of reagent and analyte. For polymerization, the reaction mixture of 98 % methyl methacrylate monomer and 2 % PEG 400 polyethylene glycol is poured into a nonreactive glass-and-silicone mold, placed into a thermostat at the temperature of 70 °C until conversion is achieved. The polymer mass obtained is a transparent product of MMA and PEG 400 polyethylene glycol copolymerization, which offers a high potential for solid-phase extraction. In this product PEG 400 shapes hydrophilic chains providing for the entry of the extractable into the polymer. The PMM plates we synthesized were 0.5 mm thick. The sensors of appropriate sizes required for the analysis were obtained by cutting the initial plates into smaller pieces 4×6 mm.

S2. Preparation of Solutions

A stock solution of TC 0.5 g/L was prepared by dissolving an accurate weight of the preparation in distilled water with the addition of 0.01 M hydrochloric acid. Working solutions with lower concentrations were obtained by diluting the stock solution on the day of the experiment. To create the necessary pH, 0.1-10.0 M HCl, 0.1 M NaOH, and standard titers for pH-metry (Ekhoskhim LLC, Russia) were used. Solutions of trichloroacetic acid 20 %, sodium borohydride 1 % and ascorbic acid 5 % were prepared by dissolving accurate weights of the preparations in distilled water. Working solutions of $HAuCl_4$ were obtained on the day of the experiment by diluting a standard solution with a concentration of 1 g/L. Reagents of qualification "chemical purity" and "pure for analysis" were used without additional purification.

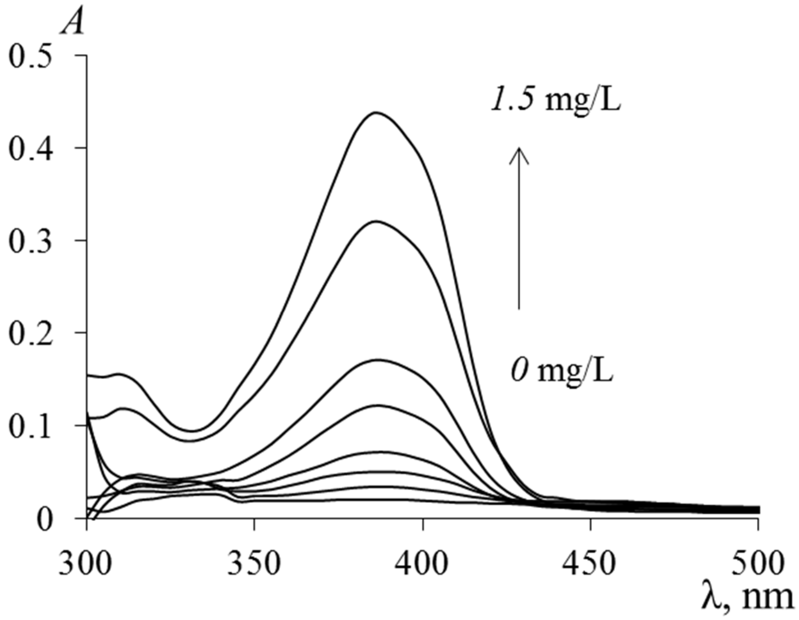

Figure S1. Absorption spectra of TC in PMM after contact with solutions of different concentrations.

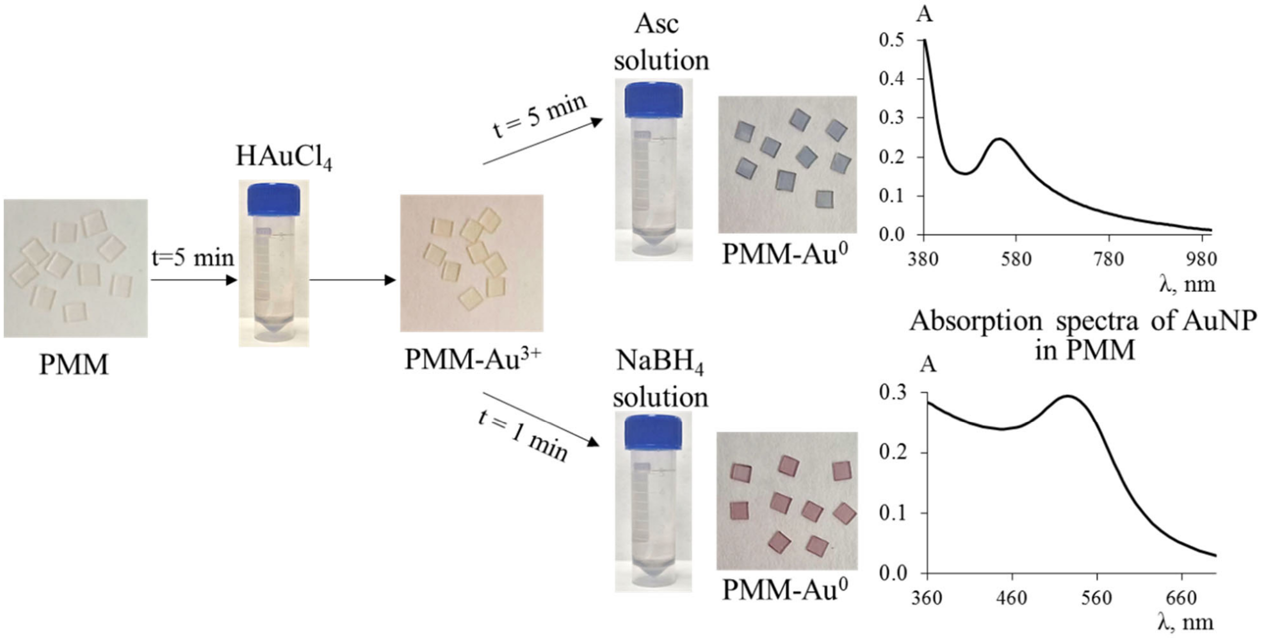

Figure S2. Scheme for obtaining gold nanoparticles in PMM.

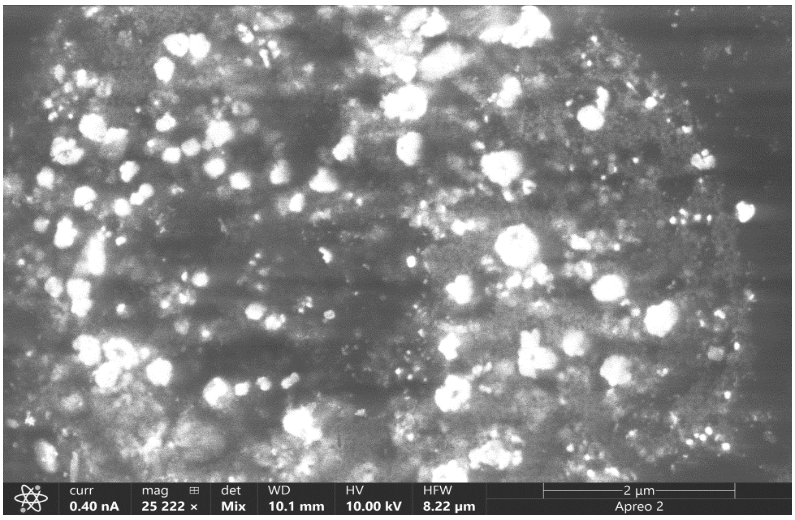

1

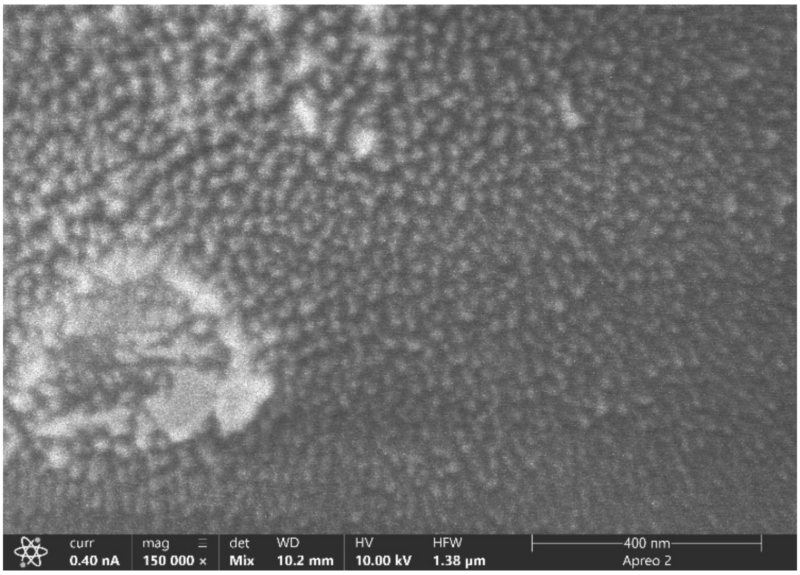

2

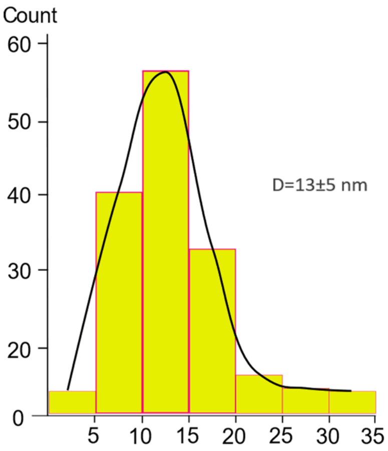

3

Figure S3. SEM images of nanoparticle associates in PMM-Au^0 (1,2) and their size distribution (3)

Table S1. Analytical characteristics of PMM-Au^0 sensors with different storage periods

Storage period	Signal	s_r	LOD, mg/L	LOQ, mg/L
2 months	*ΔE Blue*	0.04	0,0005	0,001
	ΔE Yellow-Green	0.03	0,012	0,025
	S	0.02	0,0003	0,001
4 months	*ΔE Blue*	0.05	0,0005	0,001
	ΔE Yellow-Green	0.05	0,010	0,022
	S	0.05	0,0005	0,001

Note: ΔE - Color difference in the RGB system; S - Fluorescent maximum at 520 nm.

Table S2. Comparison between our sensor and that of other jobs

Analytical system	Method	AR, mg/L	LOD, mg/L	References
PMM / PMM-Au^0	Spectrophotometry	0.05-2.0	0.03	This work
	Colorometry	0.001-2.0	0.0005	
Biometalloorganic extractant based on cobalt-gallic acid with subsequent elution of the analyte with acetonitrile and concentration by dispersed-liquid-liquid microextraction using a magnetic deep-eutectic solvent	HPLC-DAD	0.00093-1.0	0.00028	[8]
Sample preparation by magnetic solid-phase extraction (MSPE) using composite sorbent	HPLC-UV	0.001-6.0	0.0003	[9]
Isotopically labeled standard and oxalic acid extraction	of LC-MS/MS (Liquid chromatography-tandem mass spectrometry)	0.01-0.2	0.0002	[10]
SALLE extraction technology using acetonitrile as an extractant and sodium chloride for phase separation	HPLC-UV (LC-DAD) phase separation	0.06.06 - 3.0.0	0.02	[11]
Carboxyl-modified magnetic nanoparticles (CMNPs) as a sorbent for the solid-phase extraction	LC-MS/MS (Liquid Chromatography with Tandem Mass Spectrometry)	0.0001 – 0.2	$1.96 \cdot 10^{-5}$	[13]
Multicolored quantum dots with antibodies	Fluorescent enzyme-linked immunosorbent assay	(0.001 – 2.5) ·10-2^{-2}	5·10-6^{-6}	[15]
Zirconium-tetracycline complex	Spectrophotometry	0.4 – 25.0	0.13	[17]
Tetracycline-yttrium(III) complex in a cetyltrimethylammonium bromide (CTAB) micellar medium	Spectrophotometry	4-178	2.2	[19]
Carbon quantum dots doped with ammonium sulfate	Fluorimetry	0.4-44	0.05	[21]

Fluorescent Zn(II) metal-organic framework	Fluorimetry	0 – 18*	0.03	[22]
Carbon dots doped with ethylenediamine using biomass as a green precursor	Fluorometry	0.22-222	0.07	[23]
	Colorometry		0.08	
Supramolecular fluorescent sensor, constructed on the basis of human serum albumin as a recognition fragment and flavonol fluorophore as an indicator	Fluorimetry	0 – 8*	0.26	[24]
Aptamers-gold nanoparticles	Colorimetry	0.04-2.2	0.03	[5]
Nanoparticles gold	Color	measurement 0.05-0.5	0.015	[33]
